# *Letter to the Editor:* The Role of Cannabidiol in Cancer Care: Oncologist and Cancer Patient Perspectives

**DOI:** 10.1089/can.2022.0033

**Published:** 2023-03-30

**Authors:** Manan M. Nayak, Peter R. Chai, Stephanie Tung, Timothy S. Sannes, Miryam Yusufov, Ilana M. Braun

**Affiliations:** ^1^ Harvard Medical School, Boston, Massachusetts, USA.; ^2^ Department of Psychosocial Oncology and Palliative Care, Dana-Farber Cancer Institute, Boston, Massachusetts, USA.; ^3^ Phyllis F. Cantor Center for Research in Nursing and Patient Care Services, Dana-Farber Cancer Institute, Boston, Massachusetts, USA.; ^4^ Department of Psychiatry, Brigham and Women's Hospital, Boston, Massachusetts, USA.; ^5^ Department of Emergency Medicine, Brigham and Women's Hospital, Boston, Massachusetts, USA.; ^6^ Fenway Institute, Boston, Massachusetts, USA.

## Introduction

CBD is a nonintoxicating medicinal component of the cannabis plant in widespread use. A large cross-sectional survey of a predominantly North American convenience sample (*n*=2490) found over half the population using CBD medicinally, with cancer as a top 10 indication.^1^ Little is known about oncologists' clinical opinions of CBD use or how and why cancer patients use CBD medicinally. We conducted secondary analyses of quantitative and qualitative data sets to describe perceptions among both groups regarding the role of CBD in oncological care.

## Methods

The study analyzed two data sets that our research team previously described.^2,3^ Both studies were approved by the Dana-Farber Harvard Cancer Center Institutional Review Board. Protocol number (study 1: 16–361, study 2: 15–449).

*Study 1* (*n*=237) was a quantitative assessment of a nationally representative sample of oncologists to understand attitudes toward oncological medicinal cannabis. A forced-choice question asked whether participants clinically preferred “marijuana strains rich in tetrahydrocannabinol or cannabidiol.” Response options included: “rich in tetrahydrocannabinol,” “rich in [CBD],” “rich in both,” “no preference,” “I don't know,” and “I do not support medical marijuana use of any sort.”

For the current analyses, responses were collapsed into categories: rich in CBD (inclusive of “rich in both”); no preference (inclusive of not supporting “medical marijuana use of any sort”); and favoring cannabis rich in THC. Using Stata 15, Pearson's chi-square test examined statistical associations. Additional details pertaining to survey methodology are described elsewhere.^2^

*Study 2's* data set (*n*=24) comprised of semistructured qualitative interviews with participants who had physician-verified cancer diagnoses and dispensary-verified medicinal cannabis cards.^3^ Qualitative experts conducted confidential audio-recorded phone interviews using a semistructured interview guide. Deidentified transcripts were analyzed in NVivo 12 using applied thematic methods.^3^

Although the semistructured interview guide did not include a specific CBD-related question, it posed an open-ended question: “What strains (types) of marijuana do you prefer, and do you have a sense for what's in that strain?” Text matches for the terms “CBD” and “cannabidiol” were conducted for all transcripts. For every match, contiguous sections of the transcripts were reviewed by a four-person team to identify key CBD themes using applied framework analysis. Differences in interpretation were reconciled.

## Results

*Study 1.* Among the 237 oncologists who reported on their clinically preferred cannabis strains, two-thirds (66%) were male, 58% white, 80% working exclusively in adult oncology, the majority (56%) practicing in states with medicinal cannabis laws.

*Study 2.* Among the 24 cancer patients interviewed, over two-thirds (17/24) reported CBD use: of those, more than three-quarters (76%) were female and Caucasian. Almost half (47%) had some form of gastrointestinal cancer, and 41% were undergoing active cancer treatment.

### Oncologist perspective

*CBD-predominant cannabis not favored.* Although oncologists were generally open to their patients using medicinal cannabis,^2^ only 11% (27) favored cannabis rich in CBD, and 8% (19) favored cannabis rich in delta-9-THC. Eighty percent (191) reported “no preference,” “I don't know,” or “I do not support medical marijuana use of any sort” when queried about the bioactive cannabis ingredients they clinically favored.^2^ Although no significant associations existed between clinical preference for CBD and participant demographics, oncologists were more likely to favor CBD-predominant strains if they practiced in a state with legalized medicinal cannabis (df=4, *N*=226), chi-square 9.6982, *p*=0.046, or treated pediatric patients (df=8, *N*=230), chi-square 22.0569, *p*=0.005.

### Cancer patient perspective

*CBD for symptom management, as cancer-directed therapy, for general health, and to counteract delta-9-THC's mind-altering effects*. The majority of the 17 patients who endorsed using CBD did so to target symptoms, chiefly pain, but also nausea, gastrointestinal distress, poor appetite, myalgias, fatigue, sleep disturbance, and anxiety (including health related: “Forgetting is good.”). A couple of participants reported CBD to address multiple symptoms, rendering cancer treatment “manageable” and enabling reductions in conventional symptom management therapies (e.g., opioids). Seven of the 17 intended CBD for cancer-directed therapy (“CBD kills cancer”), sometimes as part of a Rick Simpson Oil protocol (a high daily cannabinoid online recipe with antineoplastic claims).

A couple posited CBD to work synergistically with delta-9-THC's antineoplastic properties. A few believed CBD to possess “restorative” qualities. An emergent theme described by seven participants was of CBD reversing delta-9-THC's mind-altering effects (“CBDs cancels out the high”). This theme was particularly prominent among participants following a Rick Simpson Oil protocol (Table 1).

**Table 1.** Exemplar Quotations from Cancer Patients Using Cannabidiol Medicinally

Major themes	Subthemes	Exemplar quotations
Cannabidiol for symptom management, as cancer-directed therapy, for general health, and to counteract delta-9-THC's mind-altering effects	Symptom management	“I don't think I could have tolerated [immunotherapy] at all had I not been taking the CBD oil.”
	Cancer-directed therapy	“It's like a one-two punch and it knocks out the cancer cells somehow.” “[CBD and delta-9-tetrahydrocannabinol] work together and they make the cancer cells kill themselves.”
	General health	“[CBD] repairing damaged DNA and repairing damaged cells” “[CBD for] whole body regeneration”
	Counteracting delta-9-THC's mind-altering effects	“I'm trying to always use that CBD oil …15 to 30 minutes after I take the [Rick Simpson Oil]. [CBD] helps prevent that high from happening. It buffers it, and so, you know, I've been getting where I'm noticing that I'm not feeling the high.” “You take too much [delta-9-tetrahydrocannabinol], CBD will counteract the effect of it.” “If you find yourself too high, you take yourself a lot of CBD oil. That instantly cuts down on the effect of the [delta-9-tetrahydrocannabinol].”
Cannabidiol, with greater social acceptability than delta-9-THC	Safer	“I'm not driving around high [on CBD].” “[CBD is a] good product because you can function.” “[CBD] allows me to function at work.” “[CBD] doesn't make me feel like the village idiot.”
	Greater social acceptability	“My last visit, I noticed that they included CBD on the list of the nutritional supplements that I take.”

*Vaping favored*. Seven of the 17 participants, unprompted, discussed their mode of CBD self-administration. Six vaped a CBD oil or distillate using an electronic device. Four consumed CBD orally and two sublingually. Most reported multiple routes of use.

*Safer, with greater social acceptability, than delta-9-THC*. A majority of participants described CBD in counterpoint to delta-9-THC. Participants viewed CBD as safer in multiple respects. Nine perceived CBD to be nonobtunding or mind altering, and, therefore, preferred for individuals reporting to work or driving. Among those who vaped, a couple reported that CBD triggered less throat irritation than delta-9-THC-predominant strains. One participant highlighted that CBD is nonaddictive; another believed CBD to be less anxiety provoking. Several participants indicated that CBD conferred less stigma due to more permissive regulations around its access, the fact that a health care professional (e.g., “a nutritionist or naturopath”) had recommended its use, and because it was more likely to be documented in their medical record.

## Discussion

These exploratory analyses uncovered a disconnect between oncologists' clinical preferences and oncology patient practices regarding CBD. Even as the majority of oncologists remained open to medicinal cannabis use, only 1 in 10 favored CBD-predominant products. By contrast, almost three-quarters of the cancer patients we interviewed used CBD, finding it useful for symptom management, cancer-directed therapy, general health, or counteracting delta-9-THC's mind-altering effects.

Participants frequently reported vaping CBD, a finding that contrasts with oncologists' known preferences for oral delivery.^4^ These preliminary findings highlight the potential need for improved bidirectional communication between cancer patients and their providers regarding the use of cannabinoids such as CBD.

Cancer patients perceived CBD, as compared with delta-9-THC, as being safer and favored by their health care teams. This finding is curious in light of our parallel finding that oncologists do not favor CBD-predominant products. Of note, physicians “don't know” responses have been previously shown to be meaningful in cannabis surveys.^5^ The discrepancy raises important questions about CBD information sources on which patients depend, particularly since our earlier study suggests that nonmedical entities, including cannabis dispensaries, are responsible for the majority of their cannabinoid advising.

Study limitations include that survey data were captured when CBD was federally illegal. Given rapid regulatory changes, sentiments among oncologists may have evolved. Patient participants comprised a small convenience sample, and their views may not fully reflect those of the national population, including cancer patients without medicinal cannabis cards. The fact that our sample was drawn from several states and that participants had physician-verified cancer diagnoses strengthened our findings.

This sociobehavioral investigation was enriched by the intersection of survey data from a nationally representative sample of oncologists and interviews with cancer patients across several states. It should be followed by rigorous national surveys of oncologists and cancer patients to better understand current CBD-related views and practices. If such surveys support our findings, then effective means of increasing patient and provider education and communication around cannabinoids such as CBD should be undertaken. Likewise, randomized oncological clinical trials with CBD are needed to identify optimal routes of administration and appropriate indications.

## Abbreviations Used

CBD: cannabidiol
THC: tetrahydrocannabinol
